# Flavonoid 8-*O*-Glucuronides from the Aerial Parts of *Malva verticillata* and Their Recovery Effects on Alloxan-Induced Pancreatic Islets in Zebrafish

**DOI:** 10.3390/molecules23040833

**Published:** 2018-04-04

**Authors:** Jung-Hwan Ko, Youn Hee Nam, Sun-Woo Joo, Hyoung-Geun Kim, Yeong-Geun Lee, Tong Ho Kang, Nam-In Baek

**Affiliations:** Graduate School of Biotechnology & Department of Oriental Medicine Biotechnology, Kyung-Hee University, Yongin 17104, Korea; hwann92@naver.com (J.-H.K.); 01030084217@hanmail.net (Y.H.N.); woojoosun88@naver.com (S.-W.J.); zwang05@naver.com (H.-G.K.); lyg629@nate.com (Y.-G.L.); panjae@khu.ac.kr (T.H.K.)

**Keywords:** *Malva verticillata*, malvaflavone A, flavonoid 8-*O*-glucuronide, zebrafish, anti-diabetes, anti-oxidant

## Abstract

*Malva verticillata* (Cluster mallow), a leafy vegetable that has been popular in East Asia for a long time, has also been used in herbal teas and medicines. The aqueous fraction of the aerial parts of *Malva verticillata*, exhibiting a very high quantity of flavonoids compared to the EtOAc and *n*-BuOH fractions, exhibited significant recovery effects on pancreatic islets damaged by alloxan in zebrafish larvae. Thus, the bioactive components responsible for this anti-diabetic activity were investigated. A new flavonoid glucuronide (**1)** and five known flavonoids were isolated from the aqueous fraction. Based on several spectroscopic methods, compound **1** was identified to be nortangeretin-8-*O*-β-d-glucuronide, and was named malvaflavone A. The A-ring of compound **1** had a 5,6,7,8-tetrahydroxy moiety, which rarely occurs in plant systems. Also 8-*O*-glucuronide attached to the flavonoid moiety was rarely occurred in plant system. Compounds **1**, **3**, **4**, and **6** significantly improved the pancreatic islet size in zebrafish at 0.1 μM, and compounds **1** and **6** were found to block β-cell K^+^ channels in experiments with diazoxide. In ABTS, ORAC, and SOD assays, compounds **1**–**5** exhibited high anti-oxidant activities compared with quercetin and BHA (positive controls), indicating that the 8-*O*-glucuronide attached to the flavonoid moiety is a key structure for the expression of anti-oxidant activity. This is the first report of the isolation of compounds **1**–**6** from *M. verticillata* as well evaluated for anti-diabetic and anti-oxidant ativities.

## 1. Introduction

Diabetes is characterized by variations in the metabolism of carbohydrates, proteins and fats. It results from insulin secretion disorders or reduced insulin sensitivity to blood sugar levels [1]. In diabetic patients and animal models, the pancreatic islet (PI) size and β-cell number are smaller than normal [2]. Protection or recovery of the PI constitution could be an efficient pharmacological approach for the treatment of diabetes. The most important route of insulin secretion is the release of insulin from islet cells, a process which is regulated by K_ATP_ channels and voltage-gated Ca^2+^ channels [3,4]. K_ATP_ channels are closed during insulin secretion, but are open when insulin release is inhibited by β-cell metabolism.

Epidemiological studies have consistently shown that regular consumption of foods such as vegetables is strongly related to a lower risk of developing chronic diseases such as diabetes [5]. Recently, consumers increasingly believe that foods contribute directly to their health [6]. Today, foods are intended not only to satisfy hunger and provide necessary nutrients, but also to prevent nutrition-related diseases and improve physical and mental well-being [7]. It is generally accepted that a food can have health-promoting properties that go beyond its traditional nutritional value. Bioactive compounds (Coms) are defined as components of food that influence physiological or cellular activity in the animals or humans that consume them. Recently, much attention has been given to bioactive food components that may be beneficial for the prevention of chronic diseases such as diabetes [8]. A considerable amount of evidence has indicated that increased oxidative damage contributes to the development of chronic diseases such as diabetes, and several epidemiologic studies have demonstrated that the consumption of polyphenol-rich vegetables is associated with a reduced risk of oxidative stress-related diseases [9,10]. Hence, there is a need to search for anti-diabetic food components that prevent chronic diseases and also promote health.

*Malva verticillata* (Cluster mallow), a leafy vegetable that has been popular in East Asia for a long time, has also been used in herbal teas and medicines [11]. Its seeds in particular have been used in traditional Chinese medicinal formulas as diuretic, laxative, and galactopoietic materials [12]. However, reports on the chemical (Chem) constituents and biological activities of the aerial parts of *M. verticillata* were very insufficient. In our preliminary study, the aqueous fraction (Fr) of the aerial parts of *M. verticillata*, exhibiting very high quantity of flavonoids comparing to the ethyl acetate (EtOAc) and *n*-butanol (*n*-BuOH) Frs [13,14]. The TLC experiment exhibited the major components of the aqueous Fr of *M. verticillata* to be oligosaccharides and flavonoids. Some literature has reported that oligosaccharides have significant anti-diabetic activity, showing a hypoglycaemic effect [15], increasing β-cell function and insulin levels [16], inhibiting α-glucosidase activity [17], and promoting peripheral tissue utilization of glucose [18]. However, it is relatively difficult to prepare the oligosaccharide Frs. Flavonoids are a group of naturally occurring polyphenolic Coms present in various fruits and vegetables, and are constituents of a number of effective traditional remedies [19]. To date, a considerable number of flavonoids have been revealed to have anti-oxidant and therapeutic effects in chronic diseases in human populations [20,21,22]. Some studies have indicated that dietary flavonoids reduce the risk of type 2 diabetes [23,24,25].

Therefore, in this study, flavonoids with anti-diabetic activity were isolated and identified from the aqueous Fr of the aerial parts of *M. verticillata*. Six flavonoids were isolated, identified, and investigated for their recovery effects on alloxan (AX)-induced PI damage in zebrafish. The recovery effects of certain Coms were found to be derived from the closing of K_ATP_ channels in PIs. The anti-oxidant potential of these flavonoids was measured with DPPH, ABTS, ORAC radical scavenging (RS), and superoxide dismutase (SOD) assays.

## 2. Results and Discussion

**Figure 1:**
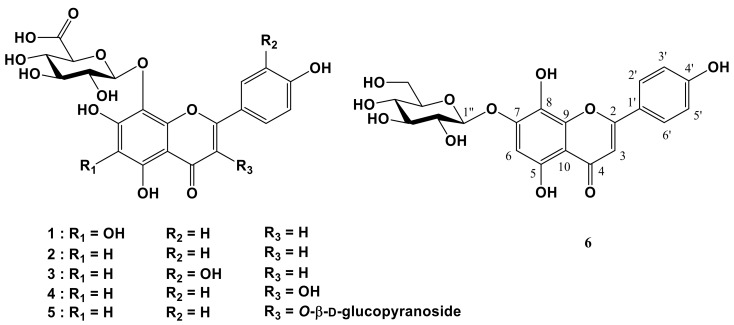
Chemical structures of flavonoids **1**–**6** isolated from the aerial parts of *Malva verticillata*.

Repeated column chromatography for the aqueous Frs of the aerial parts of *M. verticillata* resulted in the isolation of one new flavone glucuronide (**1**) and five known Coms (**2**–**6**) (Figure 1). Through spectroscopy data analyses such as IR, NMR, and ESI-QTof-MS, and through comparison of the data with those in the literature, these Coms were identified as nortangeretin-8-*O*-β-d-glucuronopyranoside (**1**), isoscutellarein 8-*O*-β-d-glucuronopyranoside (**2**) [26], hypolaetin 8-*O*-β-d-glucuronopyranoside (**3**) [26], herbacetin 8-*O*-β-d-glucuronopyranoside (**4**) [27], herbacetin 3-*O*-β-d-glucopyranosyl-8-*O*-β-d-glucuronopyranoside (**5**) [28], and isoscutellarein 7-*O*-β-d-glucopyranoside (**6**) [29]. Coms **4** and **6** have never been found in the family of Malvaceae, and coms **1**–**6** were isolated for the first time from the aerial parts of *M. verticillata* in this study.

Com **1**, a yellow amorphous powder (pyridine-*d_5_* and D_2_O), exhibited UV absorption characteristics and a pale yellow color in TLC when sprayed with 10% H_2_SO_4_ and heated. The molecular weight was determined to be 478 from the molecular ion peak *m*/*z* 479.0828 [M + H]^+^ (calculated for C_21_H_19_O_13_ 479.0826) in the positive ESI-QTof-MS. The IR spectrum suggested the presence of a hydroxyl group (3386 cm^−1^), a carbonyl group (1630 cm^−1^), a conjugated carbonyl group (1617 cm^−1^), and an aromatic double bond (1580 cm^−1^). The ^1^H-NMR spectrum contained four olefin methine proton signals (δ_H_ 8.25, 2H, d, *J* = 7.6, H-2′,6′; δ_H_ 7.35, 2H, d, *J* = 7.6, H-3′,5′), which were attributed to a paradisubstituted benzene ring B, and one olefin methine proton signal (δ_H_ 6.57, 1H, s, H-3), which was attributed to H-3 of a flavone C-ring. In the oxygen region, one hemiacetal proton signal (δ_H_ 5.05, 1H, d, *J* = 7.2 Hz, H-1′′) and four oxygenated methine proton signals (δ_H_ 4.02–4.04, H-2′′–4′′; 3.82, d, *J* = 8.8 Hz, H-5′′) were observed as the signals of a hexuronic acid moiety. The above-mentioned ^1^H-NMR spectrum indicated that Com **1** was a pentahydroxyflavone glucuronide. The ^13^C-NMR spectrum displayed 21 carbon signals, confirming that Com **1** was a pentahydroxyflavone with a hexuronic acid. One conjugated ketone carbon signal (δ_C_ 180.7, C-4), nine olefin quaternary carbon signals (δ_C_ 163.0, C-2; 161.6, C-4′; 156.0, C-9; 148.3, C-5; 144.1, C-7; 131.2, C-6; 127.9, C-8; 121.0, C-1′; 100.8, C-10), and five olefin methine carbon signals (δ_C_ 128.4, C-2′,6′; 115.7, C-3′,5′; 100.5, C-3) in the down-field area were observed as the signals of the aglycone. Based on the Chem shift of the hexuronic acid carbon signals, such as a hemiacetal (δ_C_ 101.1, C-1′′), four oxygenated methines (δ_C_ 78.6, C-3′′; δ_C_ 75.7, C-5′′; δ_C_ 74.7, C-2′′; δ_C_ 71.3, C-4′′), and one carboxyl δ_C_ 174.0 (C-6′′), the sugar was identified to be a β-glucuronic acid. From the coupling constant of the anomer proton signal (*J* = 7.2 Hz), the anomeric hydroxyl group was confirmed to be in the β-position. In the gradient heteronuclear multiple-bond connectivity (gHMBC) spectrum, the anomeric proton signal (δ_H_ 5.05, H-1′′) exhibited ^3^*J* correlation with the olefin quaternary carbon signal (δ_C_ 127.9, C-8), indicating that the β-d-glucuronopyranose was linked to C-8 of the pentahydroxyflavone. Therefore, the Chem structure of Com **1** was determined to be nortangeretin-8-*O*-β-d-glucuronide, and this Com was named malvaflavone A. The A-ring of malvaflavone A (**1**) contained 5,6,7,8-tetrahydroxy benzene, which rarely occurs in plant systems.

**Figure 2:**
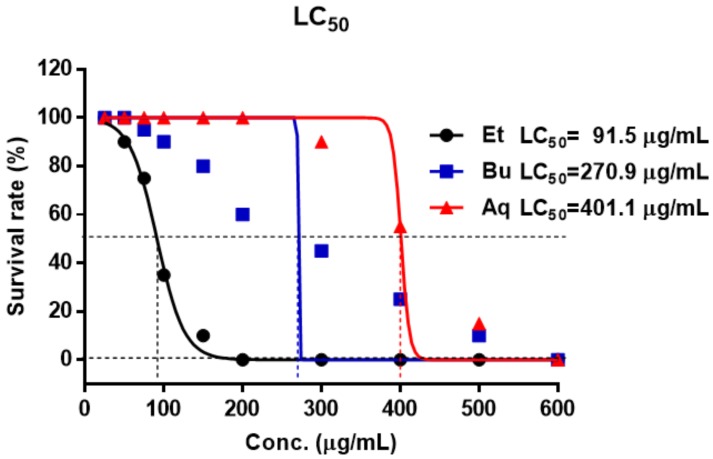
LC_50_ of zebrafish embryos exposed to EtOAc, *n*-BuOH, and aqueous Frs for 96 h. The LC_50_ of Et was 91.5 μg/mL. The LC_50_ of Bu and Aq were 270.9 μg/mL and 401.1 μg/mL, respectively. NOR: normal group, Et: EtOAc fraction, Bu: *n*-BuOH fraction, Aq: aqueous fraction.

To evaluate the toxicity of the EtOAc, *n*-BuOH, and aqueous Frs on zebrafish embryos, the survival rate related to EtOAc, *n*-BuOH, and aqueous Frs was investigated in zebrafish and LC_50_ value was calculated. Zebrafish were treated with EtOAc, *n*-BuOH, and aqueous Frs at ten different concentrations. The LC_50_ values of EtOAc, *n*-BuOH, and aqueous Frs were 91.5, 270.9 and 401.1 μg/mL, respectively (Figure 2).

**Figure 3:**
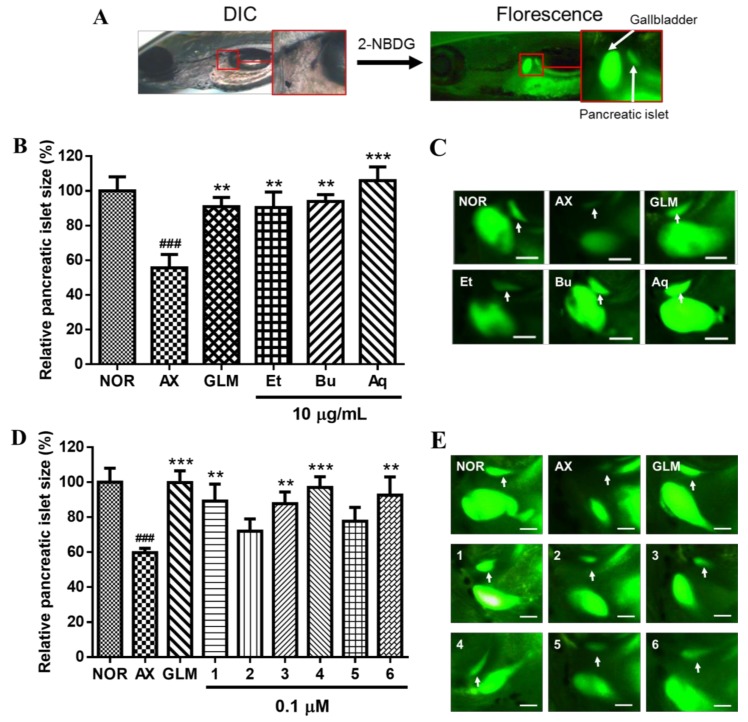
Changes in pancreatic islet (PI) area and fluorescence intensity caused by 2-NBDG. (**A**) The PI sizes of wild-type zebrafish and zebrafish larvae at 5 days post-fertilization (dpf) were evaluated by differential interference contrast (DIC) and fluorescence microscopy. (**B**) Changes in PI size caused in each fraction were analyzed with Focus Lite software. NOR: normal group, AX: alloxan-induced group, GLM: glimepiride, Et: EtOAc fraction, Bu: *n*-BuOH fraction, Aq: aqueous fraction. (^###^ *p* < 0.001; compared to NOR), (** *p* < 0.01, *** *p* < 0.001; compared to AX). (**C**) Fluorescent microscopic images of PIs treated with each fraction following treatment with 2-NBDG at 10 μg/mL. (**D**) Changes in PI size caused by compounds **1**–**6**, analyzed with Focus Lite software. (**E**) Fluorescent microscopic images of PIs treated with compounds **1**–**6** following treatment with 2-NBDG at 0.1 μM.

The isolated Coms **1**–**6** were then evaluated for their recovery effects on AX-induced PI damage in zebrafish larvae. AX is a well-known diabetogenic agent that causes a reduction of insulin release through a β-cell mass decrease. In addition, AX has already been proven in a zebrafish model in previous studies. The PI size (μm^2^) average in normal zebrafish was 1720 ± 300 μm^2^; furthermore, in AX- and glimepiride (GLM)-treated zebrafish, the average was 950 ± 200 μm^2^ and 1680 ± 220 μm^2^, respectively. PI size was 40.2% lower (*p* < 0.0001) in the AX treatment group than in the normal group (Figure 3). According to this model, after the exposure to AX, an enhanced recovery from that damage could be observed after treating the zebrafish with some Coms or extracts, the GLM is used as a positive control, since it is a well-known antidiabetic drug, and like the AX, has also been proven in the zebrafish model [30,31]. GLM was used as a positive control, as it promotes insulin secretion by closing the K_ATP_ channel [32]. Additionally, glucose uptake was assessed in the zebrafish model with 2-[*N*-(7-nitrobenz-2-oxa-1,3-diazol-4-yl)amino]-2-deoxyglucose (2-NBDG), which is a fluorescent dye derived from glucose, modified with an amino group at the C-2 position [33]. In diabetes studies, 2-NBDG is widely used to measure the ability of cells to absorb glucose [34]. Both the PI size and fluorescence intensity were significantly greater in the GLM-treated group than in the AX-treated group. When Coms **1**–**6** were added to AX-treated zebrafish larvae, Coms **1**, **3**, **4**, and **6** significantly increased the size of the injured PIs, by 49.4% (*p* = 0.0072), 46.8% (*p* = 0.0028), 62.4% (*p* < 0.0001), and 54.9% (*p* = 0.0088), respectively, compared with AX alone (Figure 3D,E). Some reports have mentioned that anti-diabetes activity was influenced in compliance with the number of hydroxyl groups in an aromatic moiety. Because Coms **1**, **3**, and **4** included one more hydroxyl group in the structure than Com **2**, the former three showed higher activity than the latter. Additionally, Com **5** has two sugar moieties in the structure, which induces the decease of the anti-diabetes activity [35,36]. Furthermore, flavonoids with 7,8-dihydroxy moiety, such as Com **6**, have been reported to improve blood insulin concentration, to lower blood glucose level, and to increase insulin sensitivity in tissues such as liver, fat, and muscles [37].

As shown in Figure 3B,D, the aqueous Fr showed higher recovery effect on the AX-induced pancreatic islets in zebrafish than those of each components. The preliminary TLC experiment indicated the aqueous Fr contained high amount of oligosaccharides, in addition to flavonoids. The oligosaccharides have been reported to have significant anti-diabetic activity in many previous works [15,16,17,18].

**Figure 4:**
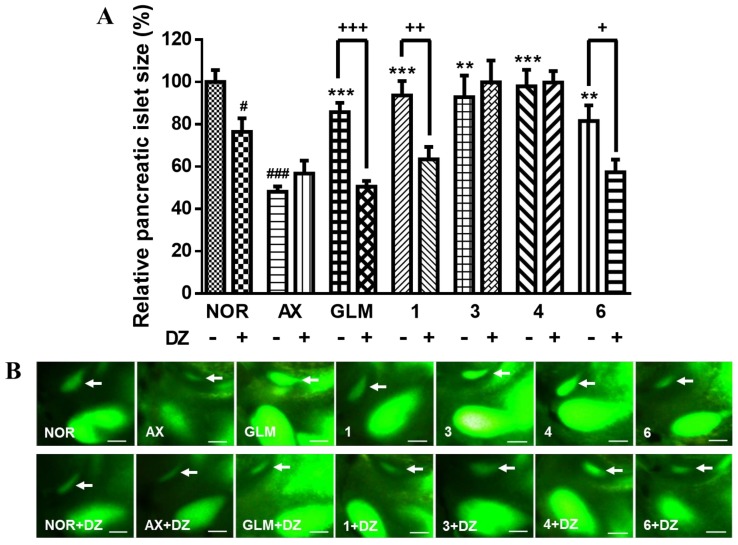
Recovery effects of flavonoids **1**, **3**, **4**, and **6** on zebrafish larvae treated with alloxan (AX) and diazoxide (DZ). Glimepiride (GLM) was used as a positive control. (^#^ *p* < 0.05, ^###^ *p* < 0.001; compared to NOR), (** *p* < 0.01, *** *p* < 0.001; compared to AX), (^+^ *p* < 0.05, ^++^ *p* < 0.01, ^+++^ *p* < 0.001). (**A**) Recovery effects of flavonoids **1**, **3**, **4**, and **6** on zebrafish larvae treated with AX and DZ. (**B**) Fluorescent microscopic images of PIs treated with compounds **1**, **3**, **4**, and **6** following treatment with 2-NBDG at 0.1 μM.

Due to their effects on PI size, Coms **1**, **3**, **4**, and **6** were investigated for their ability to alter insulin secretion by modulating K_ATP_ channels. Diazoxide (DZ) was used as a K_ATP_ channel opener, and pancreatic β-cell K_ATP_ channel stimulation activity was measured. The size of the PIs was significantly smaller (23.5%, *p* = 0.0105) in the DZ-treated normal group than in the normal group without DZ. The PI size in the AX group did not change significantly after the zebrafish larvae were treated with 25 μM DZ. The PI sizes in AX-induced zebrafish larvae treated with 0.1 μM GLM were significantly reduced following cotreatment with 25 μM DZ (41.1%, *p* = 0.0001). Similarly, in AX-induced zebrafish larvae treated with Coms **1** and **6**, the PI sizes were significantly reduced following cotreatment with 25 μM DZ (32.2%, *p* = 0.0027 and 29.7%, *p* = 0.0182, respectively). However, in AX-induced zebrafish larvae treated with Coms **3** and **4,** the PI sizes were almost the same after cotreatment with 25 μM DZ, indicating that Coms **3** and **4** had no relationship with K_ATP_ channels (Figure 4). Thus, Coms **1**, **3**, **4**, and **6** (0.1 μM) exerted significant recovery effects on the size of PIs damaged by AX in zebrafish, and Coms **1** and **6** were confirmed to influence K_ATP_ channels in DZ experiments. According to this study, the natural Coms of *M. verticillata* may exert their action by closing the K_ATP_ channels, which is demonstrated by the inhibition of the effect when cotreated with DZ, a well-known K_ATP_ channel opener, resulting in an increased insulin secretion and a consequently increased glucose uptake.

In the DPPH RS assay, Coms **3**–**5** exhibited activity comparing to positive controls (quercetin and BHA). And Coms **1**–**6** displayed significant activity, comparing to positive controls in the ABTS RS, oxygen radical absorbance capacity (ORAC), and superoxide dismutase (SOD) assays (Table 1). Coms **1**–**5** were revealed to have high anti-oxidant activities in the ABTS, ORAC, and SOD assays, indicating that the 8-*O*-glucuronic acid moiety is a key structure for the expression of anti-oxidant activity. In particular, because Coms **1** and **3** exhibited very high anti-oxidant activity, the 1,2,3-trihydroxy benzene moiety in the flavonoid A-ring and the 1,2-dihydroxy benzene moiety in the flavonoid B-ring appear to contribute to anti-oxidant activity. The glucuronic acid moieties in Coms **1**–**5** may have reduced their solubility in 70% MeOH, which was used as a solvent for the DPPH assay. Therefore, the DPPH RS activities of Coms **1**–**5** were not as high as expected. Despite low solubility of Com **3** in 70% MeOH, B-ring of Com **3** has the 1,2-dihydroxy (catechol) moiety, which is well known to exhibit high radical scavenging activity [38,39]. Com **3** showed the highest anti-oxidant activity; on the contrary, Com **6** showed the lowest activity in most assays. As mentioned above, Com **3** has the 1,2-dihydroxy (catechol) moiety. Additionally, among six flavonoid glycosides, Coms **1**–**5** have the glucuronic acid in the structure, but Com **6** has glucoside.

In this study, a new flavonoid glucuronide (**1)** and five known flavonoids (**2**–**6**) were first isolated from the aqueous Fr of the aerial parts of *M. verticillata* and identified. The 5,6,7,8-tetrahydroxy benzene ring in Com **1** was of particular interest, as it rarely occurs in plant systems. Coms **1**, **3**, **4**, and **6** (0.1 μM) were revealed to restore the PI mass in zebrafish damaged by AX treatment, and Coms **1** and **6** were found to block β-cell K^+^ channels in experiments with DZ. Additionally, Coms **1**–**5** exhibited significant anti-oxidant activity in ABTS, ORAC, and SOD assays, indicating that the 8-*O*-glucuronide attached to the flavonoid moiety is a key structure for the expression of anti-oxidant activity. Coms **1** and **3** exhibited especially high RS activities in ABTS and ORAC assays, indicating that the 1,2,3-trihydroxy benzene moiety in the flavonoid A-ring and the 1,2-dihydroxy benzene moiety in the flavonoid B-ring are closely related to anti-oxidant activity (Table 2).

In conclusion, Coms **1**–**6** were isolated from *M. verticillata*, and their anti-diabetic and antioxidant activities were evaluated for the first time. These results suggest that steady intake of *M. verticillata* could have anti-diabetic and antioxidant effects.

## 3. Experimental

### 3.1. Materials

The aerial parts of *M. verticillata* were purchased from a commercial farm in Namyangju city, Korea in April 2016. The voucher specimen (KHU20160419) is deposited at the Laboratory of Natural Products Chemistry, Kyung Hee University, Yongin, Korea. 

### 3.2. Reagents and Instrumentation

The silica gel (SiO_2_) and octadecyl SiO_2_ (ODS) resins used for column chromatography (CC) were Kiesel gel 60 (Merck, Darmstadt, Germany) and Lichroprep RP-18 (40–60 μm, Merck, Darmstadt, Germany), respectively. Sephadex LH-20 was purchased from Amersham Biosciences (Uppsala, Sweden). Thin layer chromatography (TLC) analysis was carried out with Kiesel gel 60 F_254_ and RP-18 F_254S_ (Merck) TLC plates, and the Coms were detected with a Spectroline Model ENF-240 C/F UV lamp (Spectronics Corporation, Westbury, NY, USA) and a 10% H_2_SO_4_ solution. Nuclear magnetic resonance (NMR) spectra were recorded on a 400 MHz FT-NMR spectrometer (Varian Inova AS-400, Palo Alto, CA, USA). Deuterium solvents were purchased from Merck Co. Ltd and Sigma Aldrich Co. Ltd (St. Louis, MO, USA). IR spectra were obtained on a Perkin Elmer Spectrum One FT-IR spectrometer (Buckinghamshire, England). Quadrupole time-of-flight tandem mass spectrometry (ESI-QTof-MS) spectra were recorded on a Vion IMS QTof Mass Spectrometer (Waters Corporation, Milford, MA, USA). Melting points (Mp) were obtained with a Fisher-John’s Melting Point Apparatus (Fisher Scientific, Miami, FL, USA) with a microscope, and the values obtained were uncorrected. Optical rotation was measured on a JASCO P-1010 digital polarimeter (Jasco, Tokyo, Japan). AX and sea salts were purchased from Sigma Chem Co. (St. Louis, MO, USA). GLM was obtained from Cayman Chem Co. (Ann Arbor, MI, USA). 2-NBDG was purchased from Invitrogen (Life Technologies, Grand Island, NY, USA). DZ was purchased Santa Cruz Biotechnology (Dallas, TX, USA). Fluorescence microscopy was performed on an Olympus 1X70 microscope (Tokyo, Japan). For image analysis, Focus Lite (Focus Co., Daejeon, Korea) was used.

### 3.3. Isolation of Flavonoids

Dried aerial parts of *M. verticillata* (3.1 kg) were extracted with 80% MeOH (54.0 L × 5) at room temperature for 24 h. The extracts were filtered through filter paper and evaporated under reduced pressure at 43 °C to yield 794 g of extract. The obtained MeOH extracts were suspended in H_2_O (2 L) and then successively extracted with EtOAc (2 L × 4) and *n*-BuOH (2 L × 4). Each layer was concentrated so that EtOAc (MVE, 80 g), *n*-BuOH (MVB, 75 g), and aqueous (MVW, 637 g) Frs could be obtained. The aqueous Fr (MVW, 637 g) was applied to a Diaion HP-20 column (φ 9 × 30 cm) and eluted with H_2_O-MeOH (H_2_O→5:1→3:2→2:3→1:4, 7.2 L of each) to yield nine Frs (MVW-1 to MVW-9). Fr MVW-5 [18.3 g, elution volume/total volume (V_e_/V_t_) 0.478–0.778] was subjected to ODS CC (φ 5 × 15 cm) and eluted with MeOH-H_2_O (1:4, 2.6 L) to yield nine Frs (MVW-5-1 to MVW-5-9). Fr MVW-5-4 [1.4 g, V_e_/V_t_ 0.167–0.189] was subjected to SiO_2_ CC (φ 4.5 × 15 cm) and eluted with CHCl_3_-MeOH-H_2_O (9:3:1→6:4:1, 4.4 L of both) to yield 17 Frs (MVW-5-4-1 to MVW-5-4-17). Fr MVW-5-4-13 (444.1 mg, V_e_/V_t_ 0.520–0.627) was subjected to ODS CC (φ 3 × 5 cm) and eluted with acetone-H_2_O (1:4, 1.5 L) to yield 7 Frs (MVW-5-4-13-1 to MVW-5-4-13-7). Fr MVW-5-4-13-3 [116.1 mg, V_e_/V_t_ 0.273–0.358] was subjected to SiO_2_ CC (φ 2.5 × 5 cm) and eluted with CHCl_3_-MeOH-H_2_O (6:4:1, 3.2 L) to yield 6 Frs (MVW-5-4-13-3-1 to MVW-5-4-13-3-6), which included two purified Coms, **4** and **5** [**4**, MVW-5-4-13-3-2, 24.2 mg, V_e_/V_t_ 0.200–0.369, TLC (Kiesel gel 60 F_254_) R_f_ 0.51, CHCl_3_-MeOH-H_2_O (6:4:1), TLC (RP-18 F_254S_) R_f_ 0.91, acetone-H_2_O (2:3); **5**, MVW-5-4-13-3-4, 20.5 mg, V_e_/V_t_ 0.494–0.756, TLC (Kiesel gel 60 F_254_) R_f_ 0.25, CHCl_3_-MeOH-H_2_O (6:4:1), TLC (RP-18 F_254S_) R_f_ 0.80, acetone-H_2_O (2:3)]. Fr MVW-5-4-13-4 [107.9 mg, V_e_/V_t_ 0.359–0.552] was subjected to SiO_2_ CC (φ 2.5 × 15 cm) and eluted with EtOAc-*n*-BuOH-H_2_O (4:3:1, 2.2 L) to yield nine Frs (MVW-5-4-13-4-1 to MVW-5-4-13-4-9), which included two purified Coms, **1** and **6** [**1**, MVW-5-4-13-4-6, 16.4 mg, V_e_/V_t_ 0.306–0.404, TLC (Kiesel gel 60 F_254_) R_f_ 0.90, EtOAc-isopropanol-H_2_O (1:4:1), TLC (RP-18 F_254S_) R_f_ 0.68, acetone-H_2_O (1:2); **6**, MVW-5-4-13-4-8, 28.2 mg, V_e_/V_t_ 0.557–1.000, TLC (Kiesel gel 60 F_254_) R_f_ 0.72, EtOAc-isopropanol-H_2_O (1:4:1), TLC (RP-18 F_254S_) R_f_ 0.88, acetone-H_2_O (1:2)]. Fr MVW-5-4-13-5 [86.0 mg, V_e_/V_t_ 0.553–0.670] was subjected to ODS CC (φ 2.0 × 7 cm) and eluted with MeOH-H_2_O (1:3, 2.9 L) to yield four Frs (MVW-5-4-13-5-1 to MVW-5-4-13-5-4), which included two purified Coms, **3** and **2** [**3**, MVW-5-4-13-5-1, 18.0 mg, V_e_/V_t_ 0.001–0.027, TLC (Kiesel gel 60 F_254_) R_f_ 0.50, CHCl_3_-MeOH-H_2_O (6:4:1), TLC (RP-18 F_254S_) R_f_ 0.68, acetone-H_2_O (2:3); **2**, MVW-5-4-13-5-3, 31.2 mg, V_e_/V_t_ 0.537–1.000, TLC (Kiesel gel 60 F_254_) R_f_ 0.50, CHCl_3_-MeOH-H_2_O (6:4:1), TLC (RP-18 F_254S_) R_f_ 0.62, acetone-H_2_O (2:3)].

Malvaflavone A (MVW-5-4-13-4-6, **1**), yellow amorphous powder; [α]_d_–31.9° (*c* 0.10, 50% MeOH); Mp decomposed >300 °C; IR (KBr, ν) 3386, 1630, 1617, 1580 cm^−1^; positive ESI-QTof-MS *m*/*z* 479.0828 [M + H]^+^ (calculated for C_21_H_19_O_13_: 479.0826); ^1^H-NMR (400 MHz, pyridine-*d_5_* and D_2_O, δ_H_) and ^13^C-NMR (100 MHz, pyridine-*d*_5_ and D_2_O, δ_C_); see Table 2.

Isoscutellarein 8-*O*-β-d-glucuronopyranoside (MVW-5-4-13-5-3, **2**), yellow amorphous powder; [α]_D_–11.2° (*c* 0.10, 50% MeOH); Mp decomposed >300 °C; IR (KBr, ν) 3384, 1632, 1619, 1582 cm^−1^; positive ESI-QTof-MS *m*/*z* 463 [M + H]^+^; ^1^H-NMR (400 MHz, pyridine-*d*_5_ and D_2_O, δ_H_) 8.40 (2H, d, *J* = 8.0, H-2′,6′), 7.48 (2H, d, *J* = 8.0, H-3′,5′), 6.76 (1H, s, H-3), 6.56 (1H, s, H-6), 5.07 (1H, d, *J* = 7.6, H-1′′), 4.35 (1H, m, H-4′′), 4.31 (1H, overlapped, H-2′′,3′′), 4.19 (1H, d, *J* = 8.8, H-5′′); ^13^C-NMR (100 MHz, pyridine-*d*_5_ and D_2_O, δ_C_) 181.4 (C-4), 173.8 (C-6′′), 163.8 (C-2), 160.8 (C-4′), 156.5 (C-5,7), 148.8 (C-9), 128.5 (C-2′,6′), 125.3 (C-8), 120.7 (C-1′), 115.7 (C-3′,5′), 106.1 (C-1′′), 102.6 (C-10), 101.6 (C-3), 99.4 (C-6), 76.7 (C-3′′), 75.5 (C-5′′), 73.1 (C-2′′), 71.8 (C-4′′).

Hypolaetin 8-*O*-β-d-glucuronopyranoside (MVW-5-4-13-5-1, **3**), yellow amorphous powder; [α]_D_–7.1° (*c* 0.10, 50% MeOH); Mp decomposed >300 °C; IR (KBr, ν) 3383, 1635, 1620, 1580 cm^−1^; positive ESI-QTof-MS *m*/*z* 479 [M + H]^+^; ^1^H-NMR (400 MHz, pyridine-*d*_5_ and D_2_O, δ_H_) 7.78 (1H, d, *J* = 2.0, H-2′), 7.40 (1H, dd, *J* = 8.4, 2.0, H-6′), 6.90 (1H, d, *J* = 8.4, H-5′), 6.48 (1H, s, H-3), 6.06 (1H, s, H-6), 4.51 (1H, d, *J* = 7.6, H-1′′), 3.71 (1H, m, H-4′′), 3.63 (2H, overlapped, H-2′′,3′′), 3.49 (1H, d, *J* = 8.8, H-5′′); ^13^C-NMR (100 MHz, pyridine-*d*_5_ and D_2_O, δ_C_) 182.7 (C-4), 173.8 (C-6′′), 165.7 (C-2), 159.8 (C-7), 158.3 (C-5), 151.2 (C-4′), 150.4 (C-9), 146.9 (C-3′), 130.2 (C-8), 124.1 (C-1′), 120.3 (C-6′), 114.3 (C-2′,5′), 106.1 (C-1′′), 102.7 (C-3,10), 99.5 (C-6), 76.7 (C-3′′), 75.5 (C-5′′), 73.1 (C-2′′), 71.8 (C-4′′).

Herbacetin 8-*O*-β-d-glucuronopyranoside (MVW-5-4-13-3-2, **4**), yellow amorphous powder; [α]_D_–5.0° (*c* 0.10, 50% MeOH); Mp decomposed >300 °C; IR (KBr, ν) 3380, 1633, 1622, 1579 cm^−1^; positive ESI-QTof-MS *m*/*z* 479 [M + H]^+^; ^1^H-NMR (400 MHz, pyridine-*d*_5_ and D_2_O, δ_H_) 8.74 (2H, d, *J* = 7.6, 2′,6′), 7.31 (2H, d, *J* = 7.6, 3′,5′), 6.50 (1H, s, H-6), 4.89 (1H, d, *J* = 6.8, H-1′′), 4.16 (1H, m, H-4′′), 4.05 (2H, overlapped, H-2′′,3′′), 3.90 (1H, d, *J* = 8.0, H-5′′); ^13^C-NMR (100 MHz, pyridine-*d*_5_ and D_2_O, δ_C_) 181.5 (C-4), 171.2 (C-6′′), 159.4 (C-2), 159.2 (C-4′), 156.0 (C-5,7), 147.9 (C-9), 133.0 (C-3), 131.1 (C-2′,6′), 125.5 (C-8), 120.1 (C-1′), 114.6 (C-3′,5′), 105.7 (C-1′′), 102.1 (C-10), 99.1 (C-6), 76.4 (C-3′′), 75.7 (C-5′′), 74.1 (2′′), 71.5 (C-4′′).

Herbacetin 3-*O*-β-d-glucopyranosyl-8-*O*-β-d-glucuronopyranoside (MVW-5-4-13-3-4, **5**), yellow amorphous powder; [α]_D_–5.7° (*c* 0.10, 50% MeOH); Mp decomposed >300 °C; IR (KBr, ν) 3378, 1632, 1625, 1583 cm^−1^; positive ESI-QTof-MS *m*/*z* 641 [M + H]^+^; ^1^H-NMR (400 MHz, pyridine-*d*_5_ and D_2_O, δ_H_) 8.76 (2H, d, *J* = 7.2, H-2′,6′), 7.33 (2H, d, *J* = 7.2, 3′,5′), 6.54 (1H, s, H-6), 5.79 (1H, d, *J* = 6.8, H-1′′), 4.92 (1H, d, *J* = 6.8, H-1′′′), 4.18 (1H, m, H-4′′′), 4.15 (1H, m, H-6′′a), 3.92–4.10 (4H, overlapped, H-2′′–5′′), 4.07 (2H, overlapped, H-2′′′,3′′′), 4.00 (2H, overlapped, H-6′′b,5′′′); ^13^C-NMR (100 MHz, pyridine-*d*_5_ and D_2_O, δ_C_) 182.0 (C-4), 171.7 (C-6′′′), 159.9 (C-2), 159.8 (C-4′), 156.3 (C-5,7), 148.3 (C-9), 133.5 (C-3), 131.5 (C-2′,6′), 125.9 (C-8), 120.6 (C-1′), 115.0 (C-3′,5′), 106.1 (C-1′′′), 102.8 (C-1′′), 102.6 (C-10), 99.5 (C-6), 77.4 (C-3′′), 76.7 (C-3′′′), 76.1 (C-5′′′), 76.0 (C-5′′), 74.5 (C-2′′,2′′′), 71.9 (C-4′′′), 69.8 (C-4′′), 60.9 (C-6′′).

Isoscutellarein 7-*O*-β-d-glucopyranoside (MVW-5-4-13-4-8, **6**), yellow amorphous powder; [α]_D_–19.2° (*c* 0.10, 50% MeOH); Mp decomposed >300 °C; IR (KBr, ν) 3477, 1616, 1579 cm^−1^; positive ESI-QTof-MS *m/z* 449 [M + H]^+^; ^1^H-NMR (400 MHz, pyridine-*d*_5,_ δ_H_) 8.40 (2H, d, *J* = 8.0, H-2′,6′), 7.48 (2H, d, *J* = 8.0, H-3′,5′), 6.91 (1H, s, H-6), 6.71 (1H, s, H-3), 5.56 (1H, d, *J* = 7.2, H-1′′), 4.21 (1H, dd, *J* = 12.0, 2.0, H-6′′a), 3.85–4.10 (4H, overlapped, H-2′′–5′′), 3.68 (1H, dd, *J* = 12.0, 5.2, H-6′′b); ^13^C-NMR (100 MHz, pyridine-*d*_5_, δ_C_) 182.0 (C-4), 163.8 (C-2), 161.9 (C-4′), 156.2 (C-7), 156.0 (C-5), 144.1 (C-9), 129.9 (C-2′,6′), 125.6 (C-8), 121.0 (C-1′), 115.8 (C-3′,5′), 104.7 (C-10), 102.5 (C-3), 101.2 (C-1′′), 98.7 (C-6), 77.2 (C-5′′), 75.8 (C-3′′), 73.1 (C-2′′), 69.6 (C-4′′), 60.9 (C-6′′).

### 3.4. Zebrafish Care and Maintenance

Adult zebrafish were maintained in a zebrafish system S type (1500[W] × 400[D] × 2050[H] mm) (Daejeon, Korea) and a 14 h light/ 10 h dark cycle at 28.5 °C. Two pairs of adult zebrafish were placed in a spawning box overnight to obtain zebrafish larvae. The zebrafish spawned during a 30 min period of light. Zebrafish embryos were then collected at 3 h post-fertilization for incubation, and were maintained in a 0.03% sea salt solution for a 14/10 h, light/dark photocycle in an incubator at 28.5 °C. The fish were cared for in accordance with standard zebrafish protocols approved by the Animal Care and Use Committee of Kyung Hee University (KHUASP[SE]-15-10).

### 3.5. Evaluation of the Toxicity in Zebrafish Embryo

Twenty zebrafish embryos were used treatment for toxic test. Exposure of fish embryos was performed for 96 h as outlined in the OECD TG 236 for the Fish Embryo Acute Toxicity (FET) Test [40]. 31 treatments were used: normal, EtOAc, *n*-BuOH, and aqueous Frs at the concentration 25, 50, 75, 100, 150, 200, 300, 400, 500 and 600 μg/mL, respectively. The zebrafish were observed under the microscope after 96 h treatment and dead zebrafish were recorded. LC_50_ values were calculated by non-linear regression using GraphPad Prism version 5.01 software.

### 3.6. Evaluation of Recovery Efficacy for AX-Induced PI Damage in Zebrafish Larvae

The zebrafish larvae were divided into the normal group, AX-induced group (control group), and AX-induced groups treated with the different Frs and Coms **1**–**6**. Wild-type zebrafish larvae (5 dpf) were placed into 24-well plates. The larvae were exposed to 600 μM AX for 3 h to induce PI damage. The AX-induced larvae were treated with 10 μg/mL Frs or 0.1 μM Coms for 12 h so that the recovery efficacy of the Frs and Coms could be determined. Then, the larvae were stained for 30 min with 40 μM 2-NBDG and rinsed with a 0.03% sea salt solution for 20 min. After the staining, the PIs were confirmed by fluorescence microscope and analyzed with Focus Lite software.

### 3.7. Action of DZ on AX-induced Diabetic Zebrafish

Wild-type zebrafish larvae (5 dpf) were placed in 24-well plates (10 zebrafish per well). The larvae were divided into the following 14 groups: normal, normal treated with DZ or AX, and AX-induced diabetic treated with DZ, GLM, GLM + DZ, Com **1**, Com **1** + DZ, Com **3**, Com **3** + DZ, Com **4**, Com **4** + DZ, Com **6**, and Com **6** + DZ. The Coms were applied at 0.1 μM each, and DZ was applied at 25 μM. The zebrafish larvae were treated with 600 μM AX for 3 h, after which the solution was rinsed with a 0.03% sea salt solution. The AX-induced zebrafish larvae were treated with the respective Coms and/or treatments for 12 h. Following treatment, the zebrafish larvae were stained with 40 μM 2-NBDG for 30 min and rinsed with a 0.03% sea salt solution for 20 min. After the staining, PI images were captured by fluorescence microscopy and analyzed with Focus Lite software.

### 3.8. Statistical Analysis

Statistical analysis was performed with GraphPad Prism (version 5). Data are expressed as the mean ± standard error of the mean (SEM) for three replicates. Significance was determined with repeated one-way ANOVA followed by Tukey’s test. The probability level for statistical significance was *p* < 0.05. 

### 3.9. DPPH RS Activity

According to the method of Brand-Williams [41], DPPH RS activity was measured. First, 100 µM DPPH• was dissolved in 70% aqueous methanol. Then, Coms **1**–**6** (0.1 mL) were added to 2.9 mL of the methanolic DPPH• solution. The mixture was shaken vigorously and allowed to stand at 23 °C in the dark for 30 min. The decrease in absorbance of the resulting solution was monitored at 517 nm for 30 min. The control consisted of 0.1 mL of 70% aqueous methanol and 2.9 mL of DPPH• solution. Positive controls (the natural anti-oxidant quercetin and the synthetic anti-oxidant BHA [butylated hydroxyl anisole]) were also subjected to the same procedure for comparison. The results were confirmed in triplicate analyses, and the DPPH RS activity was calculated with the following equation:

RS activity (%) = (A517_control_ – A517_sample_)/A517_control_ × 100

The EC50 was determined as the concentration required to obtain a 50% RS effect.

### 3.10. ABTS RS Activity

The ABTS RS assay was carried out according to a published protocol [42]. A radical initiator, 1.0 mM AAPH, was added to 2.5 mM ABTS in phosphate-buffered saline (PBS; pH 7.4; 0.1 M K_2_HPO_4_/KH_2_PO_4_ buffer; 150 mM NaCl). The mixed solution was heated in a water bath at 68 °C. The resulting blue-green ABTS radical solution was adjusted to an absorbance of 0.650 ± 0.020 at 734 nm with additional PBS. Then, 20 µL of the sample was added to 980 µL of the ABTS radical solution. The mixture was incubated in a 37 °C water bath under restricted light for 10 min. A control (20 µL of 70% methanol and 980 µL of the ABTS radical solution) was run with each series of samples. Positive controls (the natural anti-oxidant quercetin and the synthetic anti-oxidant BHA) were also subjected to the same procedure for comparison. The reduction of absorbance at 734 nm was measured 10 min later. The ABTS radical, exhibiting a characteristic blue-green color in its odd-electron state, loses color when its unpaired electron is paired with an electron from an anti-oxidant. The results were confirmed in triplicate analyses, and the ABTS RS activity was calculated with the following equation:

RS activity (%) = (A734_control_ – A734_sample_)/A734_control_ × 100

The EC50 was determined as the concentration required to obtain a 50% RS effect.

### 3.11. Oxygen Radical Absorbance Capacity (ORAC) Assay

The ABTS RS assay was carried out according to a published protocol [43]. Appropriately diluted samples and the standards with 150 µL of 81.6 nM fluorescein solution were added to a 96-well plate and incubated at 37 °C for 10 min with 3 min of shaking. 25 µL of 153 mM AAPH solution was added and fluorescence was then detected every minute for 90 min using a microplate reader (Infinite M200, Tecan Austria GmbH, Grödig, Austria) with 485 nm excitation and 520 nm emission wavelengths. The ORAC values were calculated according to the method of Cao et al [44], and expressed as a Trolox equivalent (μmol TE/μmol).

### 3.12. Superoxide Scavenging Activity

Superoxide RS activity was measured based on the capacity of the Coms to inhibit the photochemical production of superoxide in the riboflavin-light-NBT system. The reaction mixture for the measurement of superoxide RS activity contained 0.02 mM riboflavin, 3 mM methionine, and 0.18 mM NBT in 50 mM potassium phosphate buffer (pH 7.8). Samples in DMSO (10 µL) and 150 µL of the reaction mixture were dispensed into a 96-well plate. The reaction was started with fluorescent illumination (1000 lux) and allowed to proceed for 6 min at room temperature. The absorbance at 530 nm was measured on a microplate reader (Molecular Devices, Sunnyvale, CA, USA). The natural anti-oxidant quercetin and the synthetic anti-oxidant BHA were used as positive controls. The results were confirmed in triplicate analyses, and the superoxide scavenging activity was calculated with the following equation:

RS activity (%) = (A530_control_ – A530_sample_)/A530_control_ × 100

The EC50 was determined as the concentration required to obtain a 50% RS effect.

## Figures and Tables

**Table 1:** Antioxidant activities of flavonoids **1**–**6** on DPPH, ABTS, ORAC, and SOD assays.

Compounds	DPPH EC_50_^a^ (μM)	ABTS EC_50_ (μM)	ORAC (µmol TE/ µmol)	SOD EC_50_ (μM)
Quercetin	4.08 ± 0.36 ^b^	1.73 ± 0.09	7.33 ± 0.15	0.43 ± 0.10
BHA	8.33 ± 0.46	4.65 ± 0.18	2.86 ± 0.26	0.44 ± 0.09
**1**	>50	2.22 ± 0.05	14.38 ± 0.35	0.73 ± 0.09
**2**	>50	3.38 ± 0.15	8.06 ± 0.36	1.51 ± 0.15
**3**	5.98 ± 0.24	1.52 ± 0.04	12.48 ± 1.27	0.98 ± 0.13
**4**	31.79 ± 2.22	4.51 ± 0.13	6.56 ± 0.32	1.04 ± 0.21
**5**	33.80 ± 1.89	4.05 ± 0.14	6.42 ± 0.18	0.70 ± 0.18
**6**	>50	21.62 ± 1.26	3.83 ± 0.30	1.31 ± 0.20

^a^ EC_50_ is a concentration required to obtain a 50% antioxidant effect. ^b^ Each result was expressed as mean ± SD (μM or µmol TE/ µmol) in triplicate studies.

**Table 2:** ^1^H (400 MHz) and ^13^C (100 MHz) NMR data for compound **1** (pyridine-*d_5_* and D_2_O (5:5), δ ppm, coupling patterns, *J* in Hz) ^a^ (see Supplementary Materials).

Carbon No.	1	
	δ_c_	δ_H_
Aglycon		
2	163.0	–
3	100.5	6.57 (s)
4	180.7	–
5	148.3	–
6	131.2	–
7	144.1	–
8	127.9	–
9	156.0	–
10	100.8	–
1′	121.0	–
2′, 6′	128.4	8.25 (d, 7.6)
3′, 5′	115.7	7.35 (d, 7.6)
4′	161.6	–
Sugar moiety		
1′′	101.1	5.05 (d, 7.2)
2′′	74.7	4.02–4.04 ^b^
3′′	78.6	4.02–4.04 ^b^
4′′	71.3	4.20 (m)
5′′	75.7	3.82 (d, 8.8)
6′′	174.0	–

^a^ All proton and carbon positions were assigned by HSQC and HMBC experiments. ^b^ This signals were overlapped.

## Supplementary Materials

^1^H and ^13^C-NMR, gHSQC, gHMBC, and ESI-QTof-MS spectra data of compound **1**.
