# Supplementary material for: Flavonoid 8-O-Glucuronides from the Aerial Parts of Malva verticillata and Their Recovery Effects on Alloxan-Induced Pancreatic Islets in Zebrafish
Source: Molecules. 2018 Apr 4;23(4):833. doi: 10.3390/molecules23040833 (PMC6017522; doi:10.3390/molecules23040833)
Supplement: Supplementary file 1 [file molecules-23-00833-s001.pdf]

Supplementary Materials

Flavonoid 8-*O*-glucuronides from the aerial parts of *Malva verticillata* and their recovery effects on alloxan-induced pancreatic islets in zebrafish

Jung-Hwan Ko, Youn Hee Nam, Sun-Woo Joo, Hyoung-Geun Kim, Yeong-Geun Lee, Tong Ho Kang, Nam-In Baek*

Graduate School of Biotechnology & Department of Oriental Medicine Biotechnology, Kyung-Hee University, Yongin 17104, Republic of Korea

* Corresponding author. Tel.: +82-31-201-2610; Fax: +82-31-201-2157.

E-mail address: nibaek@khu.ac.kr (Nam-In Baek).

CONTENTS

Compounds	Data	Page
**1**	^{1}H-NMR (400 MHz, pyridine-d_5 and D_2O) spectrum	3
**1**	^{13}C-NMR (100 MHz, pyridine-d_5 and D_2O) spectrum	3
**1**	gHSQC (400 MHz , pyridine-d_5 and D_2O) spectrum	4
**1**	gHMBC (400 MHz , pyridine-d_5 and D_2O) spectrum	4
**1**	ESI-QTof-MS spectrum	5

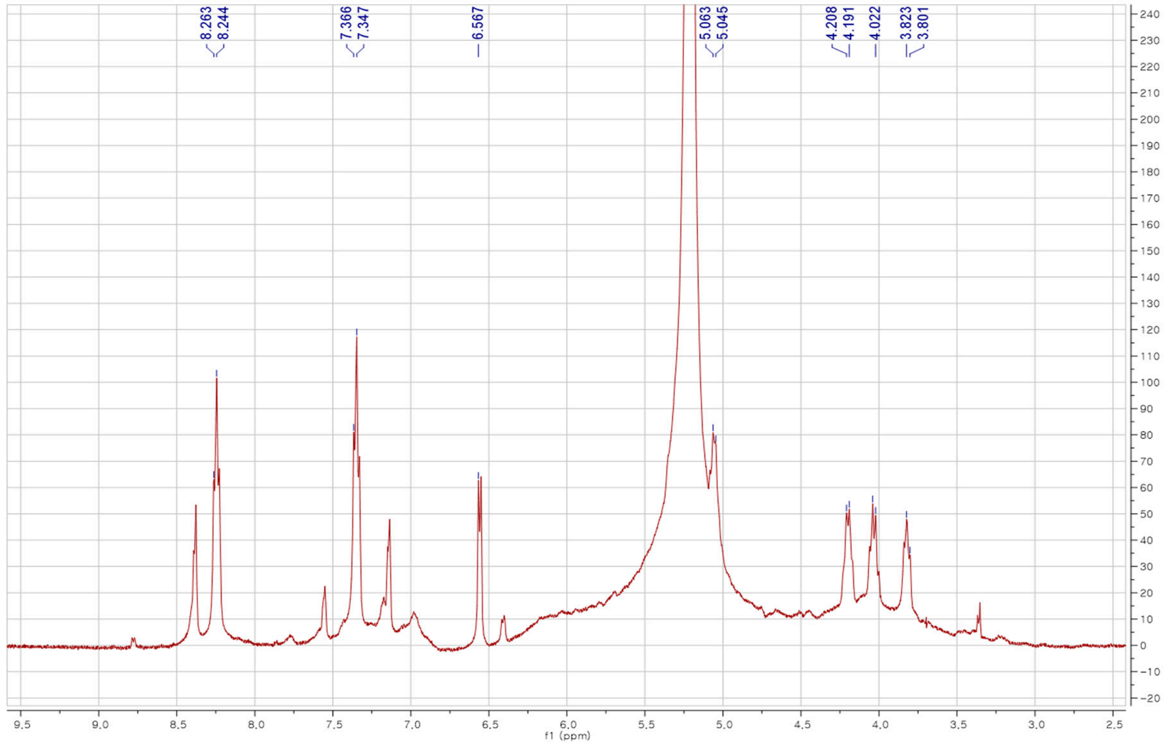

Fig. S1. ^{1}H-NMR spectrum of compound **1** (400 MHz, pyridine-d_5 and D_2O).

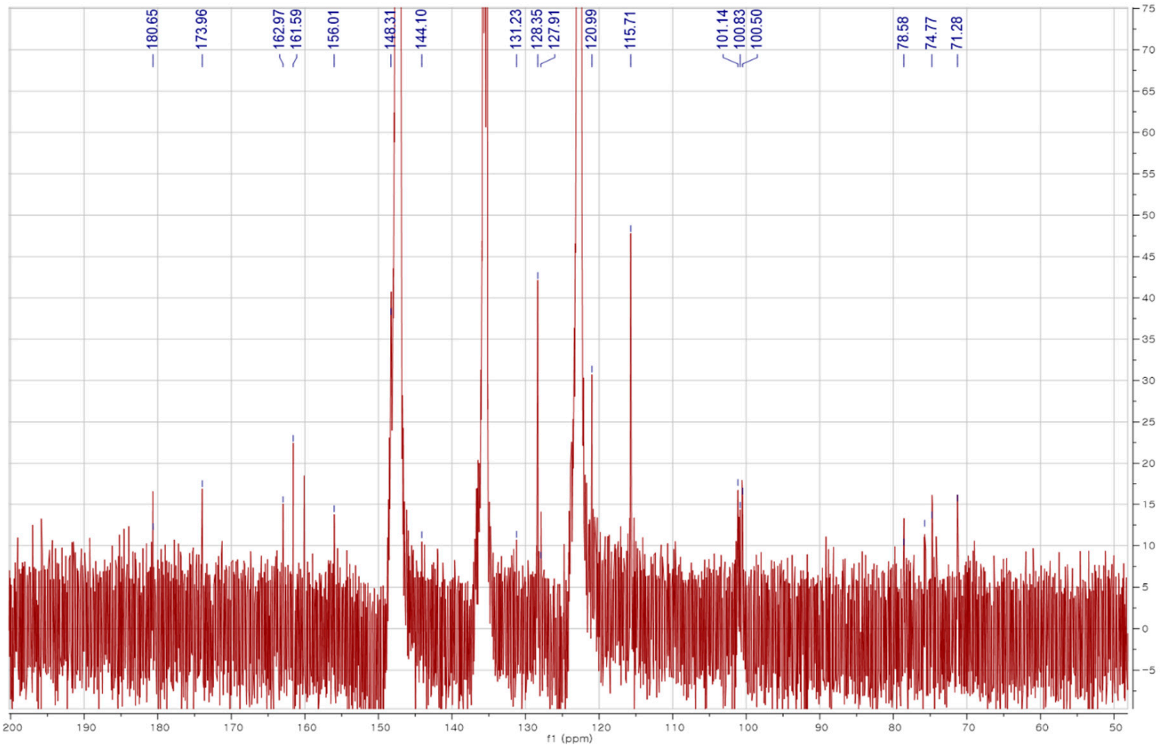

Fig. S2. ^{13}C-NMR spectrum of compound **1** (100 MHz, pyridine-d_5 and D_2O).

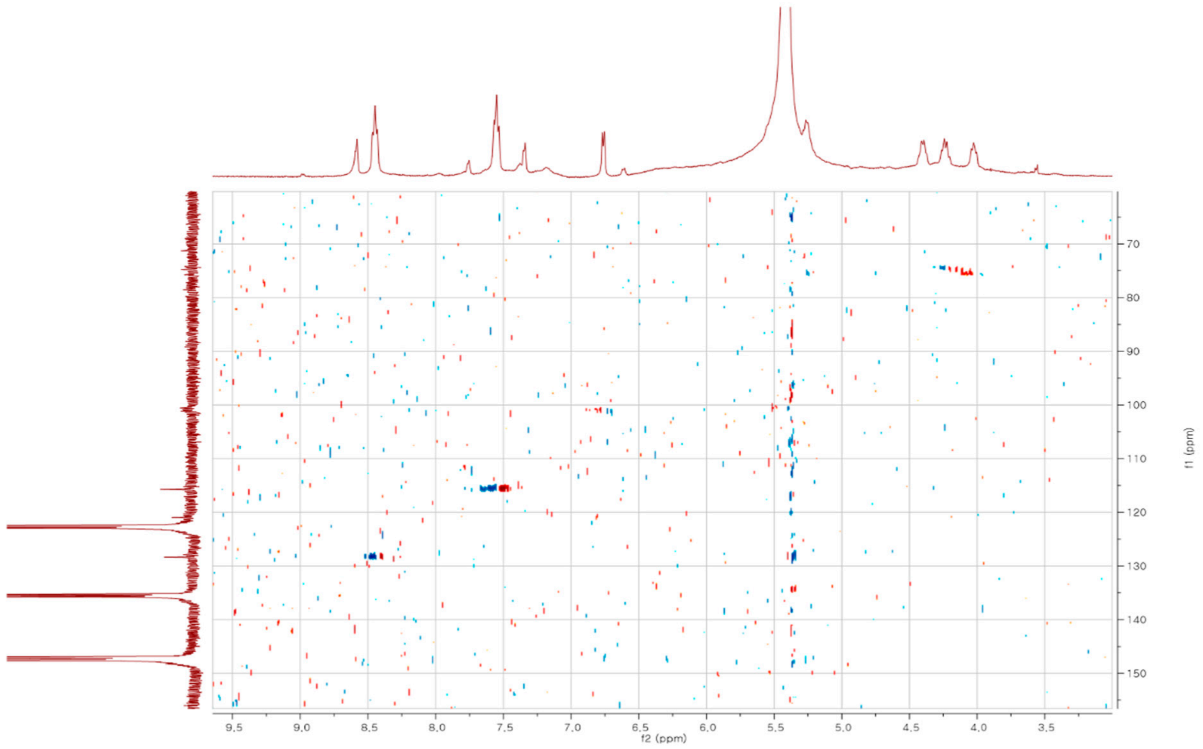

Fig. S3. gHSQC spectrum of compound **1** (400 MHz, pyridine-d_5 and D_2O).

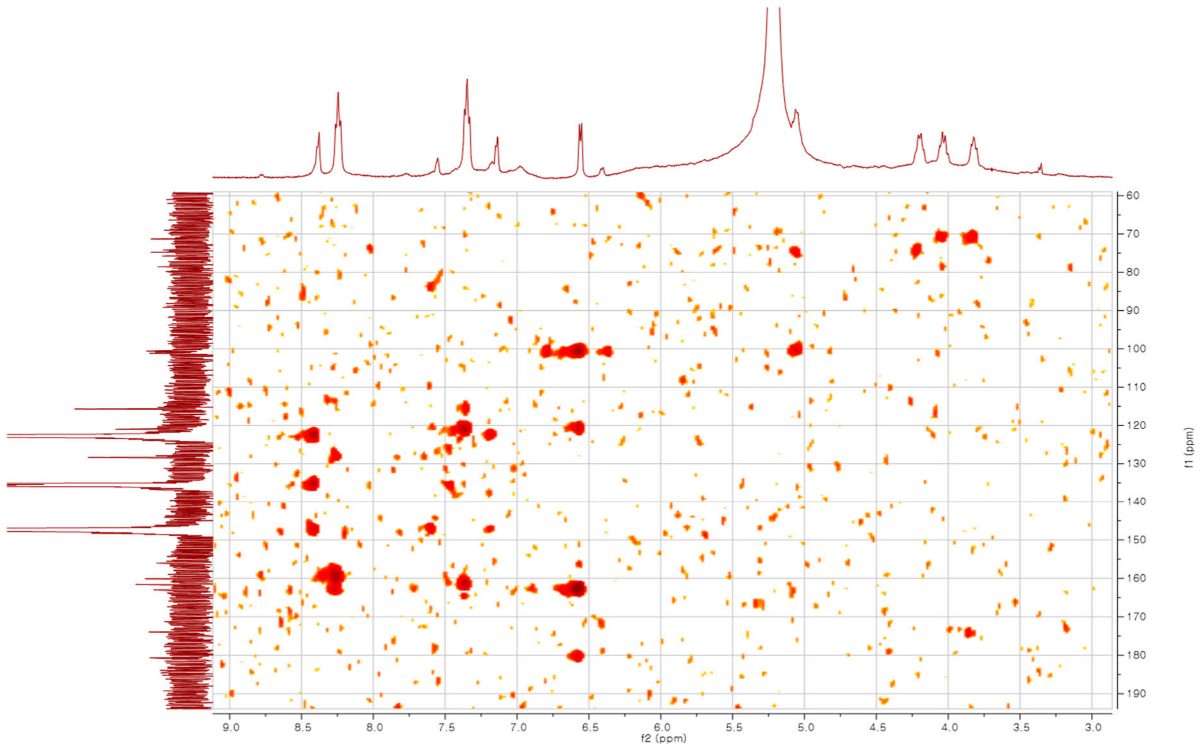

Fig. S4. gHMBC spectrum of compound **1** (400 MHz, pyridine-d_5 and D_2O).

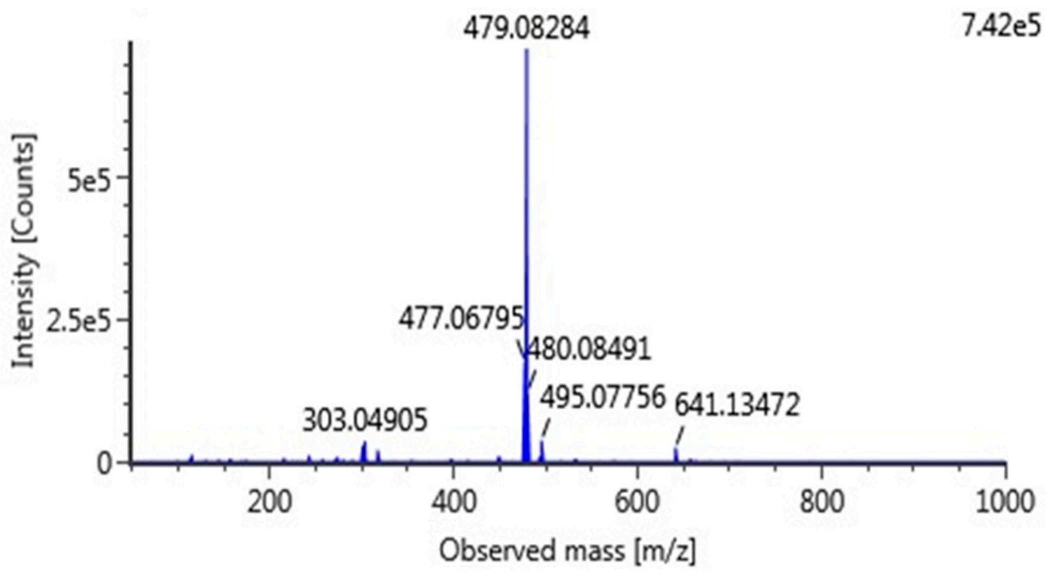

Fig. S5. ESI-QTof-MS data of compound **1**.
